# The rural-urban divide in Tanzania: Residential context and socioeconomic inequalities in maternal health care utilization

**DOI:** 10.1371/journal.pone.0241746

**Published:** 2020-11-09

**Authors:** Neema Langa, Tirth Bhatta

**Affiliations:** Department of Sociology, University of Nevada, Las Vegas, Nevada, United States of America; University of Cape Coast, GHANA

## Abstract

**Background:**

Existing studies in Tanzania, based mostly on rural samples, have primarily focused on individual behaviors responsible for the lower utilization of maternal health care. Relatively less attention had been paid to inequalities in structural circumstances that contribute to reduced utilization of maternal health care. More importantly, scholarship concerning the impact of the rural-urban divide on socioeconomic disparities in the utilization of maternal health care is virtually nonexistent in Tanzania.

**Methods:**

Drawing from the Demographic Health Survey (2015–2016) conducted in Tanzania, our study includes a total of 3,595 women aged between 15–49 years old, who had given birth in five years before the month of the interview and living in both rural and urban Tanzania. The maternal health care utilization was assessed by four variables (i.e., antenatal care, skilled delivery assistance, the before and after discharging postnatal care). The independent variables were wealth, education, residence, parity, occupation, age, and the head of the household’s sex. We used bivariate statistics and logistic regression to examine the rural-urban differences in the influence of education and wealth on maternal health care utilization.

**Results:**

Significantly lower use of maternal health care in rural than urban areas demonstrated a stark rural-urban divide in Tanzania. We documented socioeconomic inequalities in maternal health care utilization in the form of lower odds of the utilization of such services among women with lower levels of education and household wealth. The educational inequalities in the utilization of skilled delivery assistance (or = 0.37, 95% CI: 0.16, 0.86; p = 0.021) and (before discharge) postnatal care (or = 0.60, 95% CI: 0.38, 0.95; p = 0.030) were significantly wider in rural than urban areas. The differences in the odds of the utilization of skilled delivery assistance between women in poorer wealth quintile and women in richer household wealth quintile were also significantly wider in rural areas than in urban areas. However, the statistically significant rural-urban divides in the impacts of socioeconomic status on antenatal care and (after discharge) postnatal care were not observed.

**Conclusion:**

This study establishes the need for consideration of the rural-urban context in the formulation of policies to reduce disparities in maternal health care utilization in Tanzania.

## Introduction

Despite vast rural-urban differences in the distribution of health care resources in Tanzania, scant attention has been paid to their role in shaping socioeconomic inequalities in maternal health care utilization. Primarily based on rural samples, previous studies in Tanzania have mostly focused on downstream factors responsible for the lack of the usage of antenatal and skilled delivery assistance. The uncritical reliance on a range of downstream factors such as poor maternal healthcare knowledge [1], distance to health facilities [2], and knowledge about pregnancy danger signs [3], has obscured our understanding of the role of fundamental causes such as education and wealth in shaping maternal health care utilization. Moreover, existing studies in Tanzania have predominantly focused on antenatal (ANC) [2] and skilled delivery assistance (SDA) [1,4] with minimal attention to postnatal care. The postpartum period (i.e., the first six weeks after the delivery) is the most critical period with the highest risks of mortalities and morbidities for both mothers and newborns [5]. Our study draws on the Tanzania Demographic Health Survey (TDHS) to shine a light on the role of residential context in modifying the socioeconomic inequalities in MHC utilization in Tanzania.

## Rural-urban divide in health care facilities

Bereft of transformative policy changes to overhaul the centralized colonial health care system, the vast rural-urban divide in health care resources continues to exist in Tanzania. The hospitals and dispensaries that were established during the colonial era were unevenly distributed across regions, sowing the seed of inequities in MHC [6–10]. The availability of the hospital-based MHC was limited to women residing in areas of interest to colonialists. More emphasis was placed on promoting the provision of health care services in urban rather than in rural areas [6,9]. Generally speaking, during colonialism, "the center that is, the urban, and the administrative regions were developed while leaving the periphery underdeveloped" [8]. This historical legacy reverberates in the form of a rural-urban divide in health care with the disproportionate distribution of MHC in urban areas [10]. The health infrastructures that currently exist in rural areas are likely to have inadequate drug supplies [11], lack maternal health experts, and be geographically isolated [12,13]. The presence of the unequal distribution of healthcare facilities between rural and urban areas [8,14,15], and the pyramidal health care system favors urban residents than rural residents in Tanzania [16].

Despite being freely accessible, the unequal rural-urban distribution of health facilities in Tanzania is manifested in the higher utilization of ANC and SDA by women living in urban areas relative to women in rural areas [10]. In the year 2016, more women from the urban areas (63.6%) had at least four ANC visits compared to only 45.2% of rural women [17]. Eighty-seven percent of urban women used skilled delivery compared to 55% by rural women [17]. The rural-urban divide also exists in MHC services such as PNC that are not freely available. A significantly higher percentage of women from urban areas (48.0%) used postnatal care than rural women (28.9%) [17]. Given the extreme disparities in health care resources between rural and urban populations [11,18], it is surprising that very little attention has been given to the role of the residential context in modifying socioeconomic status (SES) inequalities in MHC utilization in Tanzania.

## SES inequalities in MHC utilization

The focus of prior studies, using indicators of SES such as education and wealth, has been on overall SES inequalities in MHC utilization. Drawing mostly on rural samples, such studies in Tanzania [3,7,19–25] have obtained mixed findings. Most of these studies have reported a statistically significant positive influence of wealth on the utilization of MHC. Wealthier women were more likely to utilize MHC [3,7,19,21,22,25] than their less privileged counterparts. Meanwhile, some studies have also documented statistically, not significant positive [3,23] or negative [2,20] influence of wealth on the utilization of MHC. Statistically, not significant findings could be due to differences in the number of indicators used to measure wealth.

Similar to studies on the influence of household wealth, previous research on the significance of education for MHC utilization have also reported inconsistent findings [3,19,20,23,24]. Education has been found to have a statistically significant positive influence on the use of ANC [2,25], SDA [2,3,22,25], and PNC [3,19,20]. In other words, prior studies have found the high usage of ANC, SDA, and PNC to be among individuals with higher levels of education. Some studies have also found statistically not significant positive [22,24], and negative [3] influence the utilization of ANC in Tanzania. The mixed results on the influence of wealth and education in the utilization of MHC in studies discussed may be due to inclusion of measures/variables that are related to education such as wealth and occupation, and individual behaviors that are likely to explain the relationship between education, wealth and MHC [1–3,23,24,26].

While previous studies in Tanzania have not gone beyond the assessment of overall SES inequalities in MHC utilization, scant studies in other developing countries have attempted to compare such disparities in ANC across residential context. In Ethiopia and Ghana, the influence of education and wealth on the utilization of ANC was found to be stronger for urban women than rural women. In other words, the differences in the use of ANC across levels of education and wealth were wider for urban women than rural women [27,28].

## Purpose of this study

Our study relies on fundamental cause theory [29] to examine whether the influence of education and wealth on MHC utilization differ by rural and urban residencies in Tanzania. The fundamental causes refer to material conditions such as education and wealth that place them in privileged status in terms of proximate factors such as health care utilization that is likely to shape health outcomes eventually [30,31]. More specifically, this theory acknowledges that individuals with higher SES are likely to have more excellent knowledge about and access to health care resources than their lower SES counterparts [32]. Specifically, we address the following research questions:

1. Does educational attainment influence the likelihood of MHC utilization in Tanzania?
2. Does wealth have an independent influence on the utilization of MHC in Tanzania?
3. Does the influence of wealth and education in the utilization of MHC in Tanzania vary across rural and urban residencies?

We hypothesize that women with high levels of education (secondary school and higher) are expected to utilize more ANC, SDA, and PNC than those with low levels of education. We anticipate that women in more top quintiles of household wealth are more likely to utilize the MHC than those in the lower quintiles. Finally, due to fewer numbers of health facilities with skilled health experts in MHC, we expect narrower SES inequalities in MHC in rural areas than in urban areas.

## Materials and methods

### Study population

We used the 2015–2016 Tanzania Demographic Health Survey (TDHS), which is nationally represented and administered in Tanzania. TDHS used the stratified two-stage cluster sampling design. At the first stage, an enumeration of areas is drawn from the census documents. In the second stage, samples of households are drawn to be surveyed from every enumerated area [33]. This study included a total of 3,595 married women between 15–49 years old who had given birth in five years before the interview [34] and living in both rural and urban Tanzania. Our study was also reviewed and approved by the University of Nevada, Las Vegas Social/Behavioral Institutional Review Board (IRB) on Jul 24, 2019, that the TDHS data used does not involve human subjects.

### Maternal health care utilization

MHC utilization was measured by the ANC, SDA, before discharging and after discharging PNC. The ANC was assessed by inquiring women about the number of visits they made for their ANC checkups in the health care facility. Pregnant women are recommended to visit at least four ANC visits during their pregnancy [35]. Women who had three and lower number of visits were recoded as 0, while those who had four and more visits were recoded as 1 [2,22]. The SDA was measured by asking women whether they received the delivery from health care professionals (e.g., doctors, clinical officers, assistant clinical officers, nurses or midwives, auxiliary nurses, maternal and childcare aides, as well as community healthcare workers) [22]. Women who responded yes to the care received from health care professionals were assigned 1, whereas 0 was assigned to women who did not receive such care (i.e., assisted by a non-skilled delivery attendant). The PNC was measured by asking women whether they were checked before discharge and if they were checked after release or delivery/ after giving birth at home [22].

### Socioeconomic status

Respondents’ household wealth and education status were our central explanatory variables of interest. Their education status was assessed by the level of education attained. Responses were coded into three categories, which were 0 = no education 1 = primary education and 2 = secondary education and higher (combined those with secondary school education and higher education). We treated the education variable as categorical because of the substantive significance of each level of education. The DHS rural-urban wealth index, a composite measure of household assets (e.g., availability of electricity, land ownership), was used to assess household wealth [34,36]. The DHS household wealth index was created by performing principal components analysis (PCA) of over 100 indicators of household assets [37,38]. The wealth index had five categories ranging from 1 = Poorest to 5 = Richest. For our descriptive and bivariate analysis, we used all five categories [39,40]. For our logistic regression analysis, the first two (1 = Poorest wealth quintile, 2 = Poor wealth quintile) and the last two categories (4 = Richer wealth quintile, 5 = Richest wealth quintile) were combined, which resulted in three categories (1 = Poor wealth quintile, 2 = Middle wealth quintile, 3 = Rich wealth quintile). The utilization of SDA was among the last two richer household wealth quintiles was remarkably high with virtually no variation (i.e., 97.09 for 4^th^ quintile and 100% for 5^th^ quintile). That led to convergence problems for logistic regression models that were used to predict SDA utilization. Our re-categorization of the household wealth index variable allowed us to compare regression estimates across MHC outcomes. We treated the household wealth variable as a categorical variable in our regression analysis.

### Covariates

We measured the respondents’ residential context by two categories, with 1 representing rural residence and 0 stood for the urban residence. We treated age as a continuous variable [34]. We coded occupation into three categories. Women who were not employed were recoded as 0; those informally employed (agriculture/self-employed, unskilled manual, services, household, and domestics) were recoded as 1 and formally employed (professional/ technical/ managerial, clerical, agriculture employees, and skilled manual) as 2 [41,42]. Respondents’ occupation was treated as a categorical variable. The sex of the household head was recoded 1 = female head of household and 0 for males head of household [34]. The total children ever born by women were recoded into two categories. Women with 1 to 5 children were recorded as 0 (below the total fertility rate), and women with 6 to 17 (above the total fertility rate) children were recoded as 1 [43,44]. We adopted this threshold for re-categorization because the current average fertility rate in Tanzania is 5.17 [45].

### Statistical analysis

We used STATA 15 to perform statistical analysis, which began by conducting univariate analysis (e.g., frequency, mean) to assess the distribution of each study variable [46]. We employed chi-square and t-test tests to explore the bivariate relationships between our study variables and residential context. Our analysis of the differences in SES inequalities in MHC utilization by residential setting proceeded with the calculation of absolute rate difference and rate ratios between rural and urban areas. We subtracted rates of utilization of MHC among women in urban areas from the rates for rural women. The absolutes rate difference allowed us to examine whether the likelihood of utilization of MHC is lower in rural areas across SES categories. We divided rates of utilization of MHC among rural women by corresponding rates for urban women to calculate a rate ratio. Since we used the urban as a reference, a rate ratio of less than 1 suggests a lower likelihood of utilization of MHC among rural women relative to urban women. Alternatively, a rate ratio of greater than 1 indicates a higher likelihood of utilization of MHC among rural women than urban women. We also calculated the concentration index (CI) to assess whether the distribution of MHC utilization across SES categories differs between rural and urban areas [47–49]. Our bivariate analysis was followed by regression analysis that allowed us to adjust for the influence of covariates when examining the relationship between SES (e.g., household wealth, education) and MHC outcomes (i.e., the ANC, SDA, and PNC). We addressed our first two research questions by fitting logistic regression models that adjusted for covariates (e.g., age, residence) to examine the relationship between educational attainment, wealth, and MHC outcomes. To address our third research question, we added an interaction between educational attainment and residence to assess whether the influence of education on MHC outcomes differs between rural and urban areas. We then included an interaction between household wealth and residence to explore whether the impact of wealth on MHC outcomes varied between rural and urban areas. All of our bivariate statistical tests and regression models were adjusted for multi-stage stratified sampling and non-response by using sample weights and cluster variables (primary stage units) [50].

We explored missing patterns of our variables to evaluate their potential impact in our regression estimates. Only 1.01% of respondents were missing in residence variable, and 1.78% of respondents were missing in the before discharging dependent variable. The missing indicator for all our outcomes was created (1 = missing vs. 0 = not missing). Logistic models used to predict missingness showed that our covariates were significantly related to the likelihood of missing.

## Results

The descriptive statistics presented in Table 1 show that most women (69.90%) in our study sample reside in rural areas. A higher percentage of rural women (27.20%) reported being in the richest household wealth quintile than urban women (15.62%). By contrast, a higher percentage of urban women (34.75%) reported having higher education (e.g., secondary education and higher) than rural women (15.72%). Rural women were more likely to give birth to five or more children than urban women (30.84% for rural women compared to 12.57% for urban women). Rural women were significantly more likely (74.09% vs. to 60.17% for urban women) to be informally employed. A significantly higher percentage of urban women (59.94%) had four and more ANC visits than rural women (47.33%). Similarly, a significantly higher proportion of urban women (90.67%) utilized the SDA than rural women (58.26%). Although the utilization of PNC was lower than other MHC outcomes, the rural-urban differences in the use of such care persist. Relative to 49.33% of urban women who received PNC before being discharged, only 27.15% of rural women received such care. Only 7.18% of rural women received PNC care after being discharged relative to 17.74% for urban women.

**Table 1:** Relationship between study variables and residence.

Variables	Overall Mean/%	Urban Mean/%	Rural Mean/%	Test Statistic	p-value
	(n = 3,595)	n = 2,513	n = 1082		
Education (Range 0–2)					
No education	18.55%	8.23%	23.00%	219.02	0.000
Primary school	60.00%	57.02%	61.28%		
Secondary school and higher	21.45%	34.75%	15.72%		
Wealth (Range1-5)					
Poorest	18.70%	19.50%	18.35%	66.73	0.000
Poor	19.62%	22.09%	18.55%		
Middle	18.98%	23.75%	16.91%		
Richer	19.01%	19.04%	18.99%		
Richest	23.70%	15.62%	27.20%		
Respondents’ occupation (Range 0–2)					
Not employed	18.78%	23.29%	16.83%	75.07	0.000
Informal employed	69.90%	60.17%	74.09%		
Formal employed	11.32%	16.54%	9.07%		
Age	30.62	30.99	29.88	-4.16	0.000
Number of children (1 = 5 children and more)	25.34%	12.57%	30.84%	133.44	0.000
Type of residence (1 = rural)	69.90%				
Head of household’s sex (1 = woman)	20.53%	22.74%	19.58%	4.62	0.032
Antenatal care (1 = four plus ANC visits)	51.15%	59.98%	47.35%	48.27	0.000
Skilled delivery assistance (1 = by skilled care provider)	68.01%	90.67%	58.26%	365.14	0.000
Patient checked before discharge (1 = yes)	50.17%	49.44%	27.15%	165.14	0.000
Patient checked after discharge/delivering at home(1 = yes)	14.42%	17.74%	7.28%	88.62	0.000

### Rural-urban differences in the relationship between SES and MHC utilization

Tables 2 and 3 presents results from our bivariate analysis that examines rural-urban differences in the utilization of MHC across SES groups. Negative absolute rate differences for all MHC outcomes indicated the lower utilization of such care by rural women than their counterparts in urban areas. The absolute differences in rates of utilization of SDA and (before discharge) PNC between rural and urban areas were greater than that for the other two MHC outcomes across education and household wealth categories. Besides the rate ratio of the utilization of (after discharge) PNC among women with primary education, the remaining rate ratios were less than 1. That indicates lowed utilization of MHC services by women across SES categories in rural areas relative to their counterparts in urban areas. The rural-urban gap in rates of MHC utilization was particularly notable for women with no education and primary education. The positive concentration indexes (CI) suggest that the use of MHC was mostly concentrated among women with higher education and household wealth. The concentration indexes representing educational inequalities in SDA and (before discharge) PNC utilization for rural areas (SDA CI = 0.10 and PNC CI = 0.12) was higher than urban areas (SDA CI = 0.02 and PNC CI = 0.04). The pattern was very similar for household wealth, with a higher concentration of SDA and PNC utilization among rural women with higher household wealth than urban women. We did not observe the rural-urban divide in concentration indexes for ANC and (after discharge) PNC utilization for both indicators of SES. Even though CIs representing SES inequalities in the (after discharge) PNC utilization for rural and urban areas were almost identical, they were higher than that for other MHC utilization outcomes. The rural-urban divide within SES group differences showed that such differences were generally wider in rural areas than urban areas. We observed a notable rural-urban divide for the SDA and (before discharge) PNC. For instance, the utilization of SDA among rural women with no education was 39.09% lower than those with secondary education and higher, whereas such a difference in urban areas was only 19.4%. Similarly, the utilization of SDA among rural women in the poor household wealth quintile was 40.14% lower than their counterparts in the richest household wealth quintile. The corresponding difference in urban areas was only 18.01%. The differences in the utilization of (before discharge) PNC between women with no education and secondary education were also wider in rural areas (22.56% vs. 14.43% in urban areas) than urban areas. Notably, for (after discharge) PNC, the difference in the utilization of care between women in lower SES groups and higher SES groups was wider in urban areas (e.g., the difference between no education and secondary education: 18.15% in urban areas vs. 6.87% in rural areas; the difference between poorest and riches household quintile: 19.40% in urban areas vs. 8.66% in rural areas) than rural areas.

**Table 2:** ANC AND SDA utilization disparities by respondents’ SES, Tanzania DHS (2015–2016).

	Antenatal Care				Skilled delivery assistance			
Education	Rural (%)	Urban (%)	Absolute Rate Difference	Rate ratio	Rural (%)	Urban (%)	Absolute Rate Difference	Rate ratio
No education	41.00	50.56	-9.56	0.81	40.66	75.28	-34.62	0.54
Primary school	46.36	55.59	-9.23	0.83	59.35	90.44	-31.09	0.66
Secondary school+	60.51	69.41	-8.90	0.87	79.75	94.68	-14.93	0.84
**Concentration Index**	0.06	0.06			0.10	0.02		
**Wealth**								
Poorest	42.14	45.97	-3.83	0.92	38.21	81.99	-43.78	0.47
Poorer	41.47	55.23	-13.76	0.75	48.60	86.19	-37.59	0.56
Middle	44.50	62.26	-17.76	0.71	54.27	90.66	-36.39	0.60
Rich	44.30	66.02	-21.72	0.67	61.39	97.09	-35.70	0.63
Richer	58.47	73.37	-14.90	0.80	78.35	100.00	-21.65	0.78
**Concentration Index**	0.07	0.08			0.14	0.04		

**Table 3:** PNC utilization disparities by respondents’ SES, Tanzania DHS (2015–2016).

	Before discharging				After discharging			
Education	Rural (%)	Urban (%)	Absolute Rate Difference	Rate ratio	Rural (%)	Urban (%)	Absolute Rate Difference	Rate ratio
No education	18.07	39.77	-21.70	0.45	5.54	5.62	-0.08	0.99
Primary school	27.13	47.95	-20.82	0.57	6.67	5.88	0.79	1.13
Secondary school+	40.63	54.20	-13.57	0.75	12.41	23.67	-11.26	0.52
**Concentration Index**	0.12	0.04			0.13	0.14		
**Wealth**								
Poorest	16.89	40.67	-23.78	0.42	3.71	9.00	-5.29	0.41
Poorer	24.67	46.38	-21.71	0.53	4.75	13.39	-8.64	0.35
Middle	19.90	48.24	-28.34	0.41	5.69	16.73	-11.04	0.34
Rich	26.39	52.24	-25.85	0.51	7.17	24.27	-17.10	0.30
Richer	40.60	63.10	-22.50	0.64	12.37	28.40	-16.03	0.44
**Concentration Index**	**0.17**	0.07			0.24	0.21		

### Education and MHC utilization

The logistic regression estimates presented in model 1 (see Table 4) show that even after adjusting for household wealth, the odds of the utilization of ANC among women with no education (or = 0.54, 95% CI: 0.39,0.75; p<0.001) and primary education (or = 0.61, 95% CI: 0.46,0.79; p<0.001) were significantly lower than among women with secondary education and higher. Women with no education and primary education, respectively, had 46% and 61% lower odds of utilizing ANC than women with secondary and higher levels of education. We observed similar educational differences in the utilization of (before and after discharge) PNC. The logistic regression estimates depicted in model 1 (see Table 5) illustrate that the differences in the odds of the utilization of SDA between women with lower levels of education and those with higher levels of education were the widest compared to that for other MHC services. Women with no education and primary education, respectively, had 71% and 53% lower odds of utilizing SDA than women with secondary and higher levels of education.

**Table 4:** Logistic regression estimates representing the influence of SES on antenatal care.

	Model 1		Model 2		Model 3	
Variables	OR [95% CI] (n = 3,578)	P value	OR (95% CI) (n = 3,578)	P value	OR (95% CI) (n = 3,578)	P value
Intercept	3.21[1.92,5.38]	0.000	3.29{1.93,5.63]	0.000	3.41[1.95,5.95]	0.000
Residence	0.62[0.51,0.76]	0.000	0.59[0.39,0.87]	0.008	0.56[0.36,0.86]	0.008
Head of household’s sex	0.99[0.80,1.21]	0.901	0.99[0.80,1.21]	0.905	0.99[0.81,1.22]	0.927
Age	1.01[0.99,1.02]	0.350	1.01[0.99,1.02]	0.352	1.01[0.99,1.02]	0.362
Informal employed	0.74[0.55,0.98]	0.037	0.74[0.55,0.98]	0.038	0.74[0.55,0.99]	0.042
Not employed	0.77[0.55,1.08]	0.130	0.77[0.55,1.08]	0.132	0.78[0.55,1.09]	0.139
Formal employed						
Number of children	0.75[0.57,0.98]	0.037	0.75[0.57,0.99]	0.036	0.75[0.58,0.98]	0.038
No education	0.54[0.39,0.75]	0.000	0.53[0.29,1.00]	0.049	0.61[0.32,1.17]	0.139
Primary school	0.61[0.48,0.79]	0.000	0.59[0.41,0.86]	0.005	0.64[0.43,0.95]	0.027
Secondary school+						
Poor women	0.71[0.58,0.87]	0.001	0.71[0.58,0.87]	0.001	0.58[0.38,0.88]	0.011
Middle wealth women	0.84[0.67,1.06]	0.141	0.84[0.67,1.06]	0.141	0.83[0.54,1.27]	0.378
Rich women						
Rural*no education			1.05[0.52,2.09]	0.896	0.88[0.43,1.83]	0.740
Rural*primary education			1.08[0.69,1.70]	0.736	0.98[0.60,1.59]	0.942
Rural*secondary school+						
Rural*poor women					1.34[0.84,2.14]	0.221
Rural*middle wealth					1.01[0.61,1.68]	0.966
Rural*rich women						

**Table 5:** Logistic regression estimates representing the influence of SES on skilled delivery assistance.

	Model 1		Model 2		Model 3	
Variables	OR [95% CI] (n = 3,578)	P-value	OR [95% CI] (n = 3,578)	P-value	OR [95% CI] (n = 3,578)	P-value
Intercept	41.72[20.7,84.0]	0.000	31.46[13.6,72.8]	0.000	82.9[26.8,256.2]	0.000
Residence	0.15[0.10,0.21]	0.000	0.22[0.11,0.46]	0.000	0.08[0.03,0.24]	0.000
Head of household’s sex	0.94[0.74,1.21]	0.647	0.95[0.74,1.22]	0.676	0.95[0.75,1.22]	0.712
Age	1.02[1.00,1.03]	0.046	1.02[1.00,1.03]	0.042	1.02[1.00,1.03]	0.050
Informal employed	0.72[0.47,1.12]	0.147	0.72[0.47,1.12]	0.143	0.72[0.46,1.11]	0.138
Not employed	0.54[0.33,0.90]	0.018	0.53[0.32,0.89]	0.015	0.54[0.32,0.90]	0.017
Formal employed						
Number of children	0.51[0.39,0.65]	0.000	0.51[0.40,0.66]	0.000	0.52[0.40,0.66]	0.000
No education	0.29[0.19,0.46]	0.000	0.26[0.11,0.63]	0.003	0.39[0.15,0.99]	0.049
Primary school	0.47[0.32,0.68]	0.000	0.73[0.38,1.42]	0.354	1.01[0.49,2.10]	0.971
Secondary school+						
Poor women	0.31[0.23,0.41]	0.000	0.31[0.23,0.41]	0.000	0.08[0.02,0.24]	0.000
Middle wealth	0.45[0.34,0.59]	0.000	0.45[0.34,0.59]	0.000	0.12[0.04,0.40]	0.000
Rich women						
Rural*no education			1.05[0.38,2.86]	0.930	0.66[0.23,1.90]	0.437
Rural*primary school			0.53[0.24,1.16]	0.112	0.37[0.16,0.86]	0.021
Rural*secondary school+						
Rural*poor women					4.49[1.35,14.9]	0.014
Rural*middle wealth					3.98[1.19,13.3]	0.025
Rural*rich women						

As shown in model 1 (see Table 7), the differences in the odds of (after discharge) PNC utilization between women with lower levels of education (i.e., no education and primary education) and those with secondary education and higher levels of education were not statistically significant (no education: or = 0.64, 95% CI: 0.39,1.05; p = 0.076; primary education: or = 0.77, 95% CI: 0.56,1.07; p = 0.122). Our logistic models without adjusting for household wealth (not presented in Table) indicated that the influence of education on (after discharge) PNC utilization was statistically significant. The statistically not significant estimates for education after the inclusion of household wealth in our models suggest that the influence of education on (after discharge) PNC utilization is completely mediated by household wealth. In other words, the higher utilization of (after discharge) PNC among women with higher levels of education is due to the possession of higher household wealth.

### Household wealth and MHC utilization

The logistic regression estimates presented in Model 1 (Tables 4–7) reflect the influence of household wealth on the utilization of all MHC outcomes. Women in poor household quintile had significantly lower odds of utilizing MHC than women in the rich household wealth quintile. Except for the ANC (or = 0.84, 95% CI: 0.67, 1.06; p = 0.14) and (after discharge) PNC (or = 0.71, 95% CI: 0.50, 1.01; p = 0.058), the odds of the utilization of MHC among women in middle household wealth quintile were significantly than women in rich household wealth quintile. Women in poor and middle household wealth quintile, respectively, had 29% (or = 0.71, 95% CI: 0.58, 0.87; p < .001) and 16% (or = 0.84, 95% CI: 0.67, 1.06; p = 0.14) lower odds of utilizing the 4+ ANC visits than women in rich household wealth quintile. The influence of household wealth was strongest in the utilization of SDA, followed by PNC outcomes. Women in poor and middle household wealth quintile, respectively, had 69% (or = 0.31, 95% CI: 0.23, 0.41; p < .001) and 55% (or = 0.45, 95% CI: 0.34, 0.59; p <0.001) lower odds of utilizing the SDA than women in rich household wealth quintile. Similarly, women in poor household wealth quintile had 38% (or = 0.62,95% CI: 0.50, 0.78; p <0.001) lower odds of utilization of (before discharge) PNC and 53% (or = 0.47, 95% CI: 0.32, 0.68; p <0.001) (after discharge) PNC than women in rich household wealth quintile.

**Table 6:** Logistic regression estimates representing the influence of SES on the before discharging postnatal care.

	Model 1		Model 2		Model 3	
Variables	OR [95% CI] (n = 3,515)	P- value	OR [95% CI] (n = 3,515)	P- value	OR [95% CI] (n = 3,515)	P-value
Intercept	1.08[0.59,1.96]	0.803	0.93[0.50,1.75]	0.825	0.85[0.45,1.62]	0.627
Residence	0.40[0.32,0.49]	0.000	0.60[0.39,0.93]	0.021	0.66[0.42,1.03]	0.067
Head of household’s sex	1.04[0.85,1.28]	0.680	1.03[0.86,1.27]	0.739	1.03[0.84,1.26]	0.763
Age	1.02[1.01,1.04]	0.010	1.02[1.01,1.04]	0.010	1.02[1.00,1.04]	0.009
Informal employed	0.94[0.67,1.32]	0.730	0.94[0.67,1.32]	0.721	0.95[0.68,1.33]	0.773
Not employed	0.85[0.58,1.26]	0.427	0.85[0.57,1.25]	0.404	0.84[0.57,1.24]	0.384
Formal employed						
Number of children	0.53[0.41,0.68]	0.000	0.53[0.41,0.69]	0.000	0.53[0.41,0.69]	0.000
No education	0.53[0.37,0.76]	0.001	0.65[0.34,1.26]	0.202	0.60[0.32,1.13]	0.113
Primary school	0.72[0.56,0.93]	0.011	0.89[0.66,1.19]	0.428	0.84[0.63,1.11]	0.223
Secondary school +						
Poor women	0.62[0.50,0.78]	0.000	0.62[0.49,0.78]	0.000	0.71[0.49,1.03]	0.072
Middle wealth women	0.63[0.50,0.79]	0.000	0.63[0.50,0.80]	0.000	0.80[0.57,1.12]	0.190
Rich women						
Rural*no education			0.61[0.27,1.35]	0.219	0.70[0.32,1.52]	0.363
Rural*primary school			0.60[0.38,0.95]	0.030	0.66[0.41,1.06]	0.088
Rural*secondary school+						
Rural*poor women					0.81[0.51,1.28]	0.361
Rural*middle wealth					0.65[0.40,1.06]	0.084
Rural*rich women						

**Table 7:** Logistic regression estimates representing the influence of SES on the after discharging postnatal care.

	Model 1		Model 2		Model 3	
Variables	OR [95% CI] (n = 3,578)	P- value	OR [95% CI] (n = 3,578)	P- value	OR [95% CI] (n = 3,578)	P-value
Intercept	0.42[0.17,1.03]	0.058	0.42[0.18,1.05]	0.065	0.41[0.16,1.04]	0.059
Residence	0.34[0.26,0.44]	0.000	0.32[0.18,0.54]	0	0.32[0.18,0.55]	0.000
Head of household’s sex	1.01[0.74,1.38]	0.956	1.02[0.74,1.39]	0.923	1.02[0.75,1.39]	0.918
Age	1.01[0.99,1.04]	0.386	1.01[0.99,1.04]	0.365	1.01[0.99,1.04]	0.357
Informal employed	0.60[0.44,0.83]	0.002	0.61[0.44,0.84]	0.002	0.61[0.44,0.85]	0.003
Not employed	0.56[0.36,0.89]	0.013	0.56[0.36,0.88]	0.013	0.56[0.36,0.88]	0.013
Formal employed						
Number of children	0.69[0.43,1.11]	0.129	0.69[0.43,1.11]	0.129	0.70[0.43,1.12]	0.134
No education	0.64[0.39,1.05]	0.076	0.40[1.15,1.11]	0.078	0.41[0.15,1.12]	0.081
Primary school	0.77[0.56,1.07]	0.122	0.78[0.53,1.15]	0.210	0.79[0.52,1.18]	0.245
Secondary school+						
Poor	0.47[0.32,0.68]	0.000	0.47[0.33,0.68]	0.000	0.45[0.27,0.76]	0.003
Middle wealth	0.71[0.50,1.01]	0.058	0.71[0.49,1.01]	0.055	0.76[0.47,1.23]	0.261
Rich women						
Rural*no education			1.95[0.61,6.24]	0.262	1.91[0.60,6.11]	0.274
Rural*primary school			1.00[0.53,1.87]	0.999	1.00[0.51,1.97]	0.992
Rural*secondary school+						
Rural*poor women					1.10[0.54,2.24]	0.796
Rural*middle wealth					0.80[0.38,1.67]	0.551
Rural*rich women						

### Rural-urban differences in the influence of SES on MHC utilization

The logistic regression estimates presented in Tables 4–7 show that the rural-urban divide in the utilization of all MHC outcomes exists even after the adjustment for SES and demographic characteristics. The odds of utilizing ANC, SDA, (before discharge) PNC, and (after discharge) PNC was significantly lower among rural women and urban women. Model 2 and 3 (see Tables 4–7) also suggest the rural-urban divide in the influence of SES on the utilization of MHC. The differences in odds of utilizing ANC between women with lower levels of education (no education and secondary education) and higher levels of education (secondary education and higher) were wider in rural than urban areas, but such rural-urban divide, as represented by the interaction effects (No education: or = 0.88, 95% CI: 0.43, 1.83; p = 0.74; Primary Education: or = 0.98, 95% CI = 0.60, 1.59; p = 0.94) were not statistically significant. Although narrower in rural than urban areas, the differences in the odds of the utilization of ANC between women in poorer household wealth quintile (i.e., poor and middle household wealth quintile) and richer household wealth quintile were not statistically significant (Poor wealth quintile: or = 1.34, 95% CI: 0.84, 2.14, p = 0.22), and (Middle wealth quintile: or = 1.01, 95% CI: 0.61, 1.68, p = 0.97). The rural-urban divide in SES inequalities in the utilization of (after discharge) PNC was also not statistically significant.

Model 3 in Table 5 shows that the education and household wealth-related inequalities in the utilization of SDA were significantly different between rural and urban areas. We observed a significant rural-urban divide in the difference in odds of the utilization of SDA between women with primary education and those with secondary education. The difference in the odds of utilizing SDA between women with primary education and those with secondary education was significantly wider (or = 0.37, 95% CI: = 0.16, 0.86, p = 0.021) in rural areas than in urban areas. The differences in the odds of the utilization of SDA between women in poor and middle household wealth quintiles and women in rich household wealth quintiles were also significantly narrower in urban areas than in rural areas. Finally, the difference in the odds of utilizing (before discharge) PNC between women with primary education and those with secondary education was significantly wider (or = 0.60, 95% CI = 0.38, 0.95, p = 0.03) among women in rural areas than their counterparts in urban areas.

## Discussion

This study engaged in the first systematic investigation of rural-urban differences in SES inequalities in a comprehensive set of MHC outcomes in Tanzania. Unequal distribution of health care facilities between rural and urban areas in Tanzania warranted an investigation on the role of residential context in shaping SES inequalities in the utilization of MHC. Prior scholarships in Tanzania have predominantly focused on downstream factors such as individual behaviors responsible for the lower use of a range of MHC outcomes [1,7,21]. Limited numbers of previous studies that examine SES inequalities in MHC have relied primarily on rural or non-representative samples [3,7,19–21,23,24]. Consistent with existing studies [15,17,27,51–53], our study documented a significant rural-urban divide not just in the utilization of MHC, but also in SES inequalities in the utilization of such care. Even after the adjustment for SES and demographic characteristics, this study found a significantly lower usage of MHC among rural women than their counterparts in urban areas [17]. The lower utilization of MHC in rural areas reflects the unequal distribution of health facilities and transportation services between rural and urban areas [8,16]. Despite the rural-urban divide, it was notable that very few women in both rural and urban areas utilized (after discharge) PNC. Due to costs associated with the PNC, access to such care is beyond the reach of many women, especially those residing in rural Tanzania [20,54].

Consistent with fundamental cause theory, our findings showed greater utilization of MHC by women in privileged SES as represented by higher levels of education and household wealth [29,31,55]. Our results did not align with several previous studies that reported statistically not the significant influence of SES on the utilization of MHC outcomes [22–24]. The findings, however, confirmed our hypotheses regarding the impacts of SES on MHC outcomes [2,3,19–22,25], which anticipated greater utilization of ANC, SDA, and PNC among women with higher levels of education and wealth quintiles than their counterparts with lower levels of education and wealth quintiles. Except for the PNC, which is provided free of charge in Tanzania, the lower utilization of MHC among women with lower SES reflects a lack of knowledge about the benefits of MHC services. Also, this scenario suggests other structural challenges, such as access to transportation services and a corrupt health care system that could lead to inequalities in MHC outcomes. It is common for hospitals in Tanzania to be without essential supplies for delivery [56]. As a result of the deficiencies in essential supplies for delivery, pregnant women asked and obliged to go with all required supplies such as gloves, scissors, razor blades whenever they want to give birth [7,56]. Women, especially those with lower levels of education and wealth, could be discouraged from accessing care because of other administrative costs such as financial support to the community health workers [35].

We also observed that the influence of SES varied by MHC outcomes with notably greater SES inequalities in SDA. Despite the ANC and SDA being free of charge [14,57], during delivery, the expectant mothers are subjected to unfair billings [58] and unofficial administrative charges [59], and transportation costs [10,56]. In effect, there are indirect and uncovered costs of SDA. In such a context, the educated women and those in the highest wealth quintiles are likely to have salaried jobs and financial assets that afford them means of transportation to access institutionalized care when they are in labor [30,60–62].

Findings regarding the rural-urban divide in the influence of SES on MHC did not align with our hypothesis, which expected narrower SES inequalities in such care in rural than urban areas. Scant previous studies in other developed countries have documented wider educational differences in ANC in urban than rural areas [17]. By contrast, we recorded wider educational disparities in the utilization of ANC and SDA in rural than urban areas. Similarly, the educational inequalities in PNC were wider in rural than in urban areas. In other words, the effect of SES on SDA and PNC before discharge was significantly greater for women in rural areas than their counterparts in urban areas. The rural-urban divide in the educational inequalities in the utilization of SDA and PNC suggests that education could be more salient in residential contexts that lack health care resources. Women who reside in rural areas are confronted with the reality of fewer health facilities. In such a setting, education could provide women living in rural areas with the necessary knowledge about health services and socioeconomic resources to navigate available health care resources in those areas [30,63].

Given that urban women are more likely to have access to healthcare facilities and MHC experts, the educational inequalities in the utilization of such care may be narrower than that for rural women [10]. We also found the influence of household wealth on SDA to be significantly stronger in rural than in urban areas. This finding does not align with a previous study [27] that documented a greater effect of household wealth on ANC in urban than rural areas. Our findings reflect the potential impacts of fewer numbers of health facilities with health experts skilled in MHC in elevating the role of household wealth in increasing the utilization of SDA among women in rural areas relative to those residing in urban areas.

## Study limitations and implications

Despite significant substantive contributions, this study is not without limitations. By focusing only on married women, the findings may not be generalizable to women who have other types of marital statuses. Future studies should attempt to include women of different matrimonial states to provide more comprehensive information about SES inequalities in MHC in Tanzania. Furthermore, this analysis focused only on the maternal aspect of PNC. This study could be extended to include the examination of the influence of SES on the utilization of the newborn PNC in Tanzania. Even with limitations, our study significantly advances prior scholarship that tends to focus only on the rural-urban divide in the use of MHC, with virtually no attention to such a split in the SES inequalities in the care. This extension of scholarship encourages policymakers to be mindful of the residential context in designing policies aimed to reduce SES inequalities in MHC.

The existence of SES inequalities suggests the urgent need to focus not only on the provision of free MHC but also on supporting young girls and women to attain higher education. Tanzania is one of the countries that forcibly expels young pregnant or married girls out of school when they are found pregnant [64]. Other countries include Sierra Leone and Equatorial Guinea [64]. Eight thousand young girls are dismissed out of schools every year in Tanzania [65]. Our findings highlight the need for policies that do not discriminate against pregnant girls’ access to school but empowering women to pursue their higher education by removing institutional barriers that women, especially pregnant girls, face.

Additionally, future public policies should encourage facilitating the private ownership and power of controlling the household assets for women aged above 18 years old. Only through the eradication of education and wealth differences in developing countries like Tanzania we can promote equity in the health of women during pregnancy, birth, and after birth. The wider SES inequalities in the utilization of MHC services in rural areas suggest the need to enact policies that increase the availability of health care resources to those areas. Also, future policies need to address structural barriers (e.g., access and affordability of transportation services) that limit the ability of rural women to access MHC even when they are free (e.g., SDA). Finally, findings highlight an urgent need to make PNC freely available because that is likely to encourage higher utilization of the care and eventually reduce SES inequalities in MHC, especially among rural women.

## Conclusion

Our study documented statistically significant overall influences of education and household wealth on the utilization of MHC in Tanzania. The odds of the utilization of MHC was significantly greater among women with higher levels of education and household wealth than their lower SES counterparts. Among all MHC outcomes, the SES inequalities in the utilization of SDA was the widest. The statistically significant lower odds of the utilization of MHC among rural women than urban women were observed even after the adjustment for demographic and socioeconomic characteristics. The rural-urban divide also extended to SES inequalities in the utilization of MHC. The educational disparities in the usage of SDA and (before discharge) PNC were significantly wider in rural than urban areas. The household wealth-related inequalities in the utilization of SDA were wider (i.e., the effects of household wealth were stronger) in rural than urban areas.

## Supporting information

S1 Data (XLS)
