# Correction: The rural-urban divide in Tanzania: Residential context and socioeconomic inequalities in maternal health care utilization

**DOI:** 10.1371/journal.pone.0288419

**Published:** 2023-07-06

**Authors:** Neema Langa, Tirth Bhatta

There are errors in Tables 1–7. In Table 1, an increase in the sample size has been documented. The overall sample increased from 3,595 to 7,050. The rural women sample increased from 1,082 to 1,837. The urban sample increased from 2,513 to 5,213. All the other substantive interpretations remained the same despite some increase in means or percentages. In Tables 2 and 3, despite the changes in the percentages, rate ratios, and concentration indexes of antenatal, skilled delivery, before (discharging) postnatal care, and after (discharging) postnatal care, the substantial interpretation of the concentration index, rate ratios, and absolute differences, remain the same. In Table 4, despite some minor deviation in the odds ratios, they are still statistically not significant. The odds ratios of rural women with no education likelihood of utilizing antenatal care changed from (or = 0.88 [CI = 0.43,1.83] p>.05) to (or = 1.22 [CI = 0.71,2.10] p>.05). The odds of the rural women with primary school education changed from (or = 0.98 [CI = 0.60,1.59] p>.05) to (or = 1.08 [CI = 0.76,1.52] p>.05). In Table 5, while the odds ratios remained statistically not significant, some minor deviation in odds ratios were observed. The odds changed from (or = 1.01 [CI = 0.49,2.10] p>.05) to (or = 0.81 [CI = 0.48,1.38] p>.05). In Table 6, the odds of utilizing the before (discharging) postnatal care among rural women with primary education are still low, but the level of significance changed from being weakly significant (or = 0.06[CI = 0.38,0.95]p = 0.030) to being not statistically significant (or = 0.82[CI = 0.59,1.12]p>.05). The odds ratios of utilizing the before (discharging) postnatal care among rural poor women changed from (or = 0.81 [CI = 0.51,1.2] p>.05) to (or = 1.06 [CI = 0.73,1.53] p>.05), however the results in both cases are not statistically significant. In Table 7, there were no substantial changes in the table, despite some minor deviation in the odds ratios. Please see the correct Tables 1–7 here.

**Table 1:** Relationship Between Study Variables and Residence.

	Overall Mean/%	Urban Mean/%	Rural Mean/%	Test Statistic	P-value
	(n = 7,050)	(n = 1,837)	(n = 5,213)		
Education (Range 0–2)					
No education	19.59%	8.49%	23.42%	454.85	0.000
Primary school	60.60%	56.34%	62.09%		
Secondary school and higher	19.87%	35.17%	14.48%		
Wealth (Range1-5)					
Poorest	20.65%	3.21%	27.60%	60.89	0.000
Poor	19.25%	7.08%	27.87%		
Middle	18.94%	10.07%	19.59%		
Richer	19.05%	24.66%	17.26%		
Richest	22.11%	54.98%	7.67%		
Respondents’ occupation (Range 0–2)					
Not employed	16.75%	23.73%	14.29%	196.81	0.000
Informal employed	73.29%	60.91%	77.65%		
Formal employed	9.96%	15.35%	8.06%		
Age	29.28	28.91	29.42	-2.65	0.004
Number of children (1 = 5 children and more)	22.11%	11.59%	25.84%	159.59	0.000
Type of residence (1 = rural)	73.94%				
Head of household’s sex (1 = female)	17.90%	21.34%	16.64%	19.99	0.000
Antenatal care (1 = four plus ANC visits)	49.67%	60.62%	45.81%	118.57	0.000
Skilled delivery assistance (1 = by skilled care provider)	67.28%	88.95%	59.64%	50.33	0.000
Patient checked before discharge (1 = yes)	33.29%	48.59%	27.86%	259.02	0.000
Patient checked after discharge/delivering at home(1 = yes)	14.10%	19.05%	12.35%	530.05	0.000

**Table 2:** ANC AND SDA utilization disparities by respondents’ SES, Tanzania DHS (2015–2016).

	Antenatal Care				Skilled delivery assistance			
Education	Rural (%)	Urban (%)	Absolute Rate Difference	Rate ratio	Rural (%)	Urban (%)	Absolute Rate Difference	Rate ratio
No education	39.26	47.71	-8.45	0.82	43.16	69.23	-26.07	0.62
Primary school	45.86	57.77	-11.91	0.79	60.36	88.89	-28.53	0.68
Secondary school+	56.18	68.27	-12.09	0.82	83.18	93.81	-10.63	0.89
**Concentration Index**	0.05	0.05			0.10	0.03		
**Wealth**								
Poorest	36.31	49.14	-12.83	0.74	42.39	81.99	-39.6	0.52
Poorer	43.23	54.4	-11.17	0.79	52.2	86.19	-33.99	0.61
Middle	42.76	65.51	-22.75	0.65	55.7	90.66	-34.96	0.61
Rich	45.87	66.67	-20.8	0.69	63.65	97.09	-33.44	0.66
Richer	57.82	70.75	-12.93	0.82	79.37	100	-20.63	0.79
**Concentration Index**	0.07	0.07			0.10	0.04		

**Table 3:** PNC utilization disparities by respondents’ SES, Tanzania DHS (2015–2016).

	Before discharging				After discharging			
Education	Rural (%)	Urban (%)	Absolute Rate Difference	Rate ratio	Rural (%)	Urban (%)	Absolute Rate Difference	Rate ratio
No education	18.11	35.71	-17.6	0.51	9.66	12.18	-2.52	0.79
Primary school	28.36	46.05	-17.69	0.62	11.58	16.14	-4.56	0.72
Secondary school+	41.31	55.84	-14.53	0.74	20	25.39	-5.39	0.79
**Concentration Index**	0.01	0.06			0.11	0.13		
**Wealth**								
Poorest	18.83	36.61	-23.78	0.51	8.90	9.73	-0.83	0.91
Poorer	25.16	46.4	-21.71	0.54	10.34	16.58	-6.24	0.62
Middle	24.06	49.63	-28.34	0.48	11.4	18.77	-7.37	0.61
Rich	28.19	54.87	-25.85	0.51	12.85	25.65	-12.8	0.50
Richer	40.03	59.45	-22.5	0.67	17.31	27.89	-10.58	0.62
**Concentration Index**	**0.11**	0.08			0.11	0.17		

**Table 4:** Logistic Regression Estimates Representing the Influence of SES on Antenatal Care.

	Model 1		Model 2		Model 3	
Variables	OR [95% CI] (n = 7,019)	P value	OR[95%CI] (n = 7,019)	P value	OR (95% CI) (n = 7,019)	P-value
Intercept	1.89[1.31,2.74]	0.001	2.00{1.35,2.96]	0.001	2.03[1.36,3.03]	0.001
Residence	0.55[0.47,0.64]	0.000	0.48[0.36,0.64]	0.000	0.47[0.34,0.64]	0.000
Head of household’s sex	1.13[0.97,1.31]	0.115	1.13[0.98,1.31]	0.103	1.13[0.98,1.31]	0.094
Age	1.02[1.01,1.03]	0.001	1.02[1.01,1.03]	0.001	1.02[1.01,1.03]	0.001
Informal employed	0.83[1.68,1.02]	0.075	0.83[0.68,1.02]	0.081	0.84[0.68,1.03]	0.098
Not employed	1.00[0.79,1.28]	0.993	1.01[0.79,1.28]	0.951	1.01[0.79,1.29]	0.928
Formal employed						
Number of children	0.63[0.53,0.81]	0.000	0.62[0.52,0.77]	0.000	0.63[0.52,0.77]	0.038
No education	0.65[0.58,0.75]	0.000	0.51[0.32,0.84]	0.008	0.58[0.35,0.96]	0.034
Primary school	0.78[0.66,0.92]	0.004	0.73[0.56,0.95]	0.021	0.77[0.52,1.02]	0.069
Secondary school+						
Poor women	0.64[0.56,0.75]	0.000	0.65[0.56,0.75]	0.000	0.55[0.41,0.73]	0.000
Middle wealth women	0.81[0.69,0.95]	0.008	0.80[0.68,0.94]	0.007	0.88[0.64,1.20]	0.407
Rich women						
Rural*no education			1.40[0.83,2.36]	0.205	1.22[0.71,2.10]	0.463
Rural*primary education			1.14[0.82,1.59]	0.423	1.08[0.76,1.52]	0.677
Rural*secondary school+						
Rural*poor women					1.26[0.61,1.26]	0.186
Rural*middle wealth					0.87[0.61,1.26]	0.474
Rural*rich women						

**Table 5:** Logistic Regression Estimates Representing the Influence of SES on Skilled Delivery Assistance.

	Model 1		Model 2		Model 3	
Variables	OR [95% CI] (n = 7,050)	P-value	OR [95% CI] (n = 7,050)	P-value	OR [95% CI] (n = 7,050)	P-value
Intercept	36.22[20.1,65.2]	0.000	29.34[15.5,55.9]	0.000	58.22[27.7,122.4]	0.000
Residence	0.20[0.14,0.30]	0.000	0.28[0.17,0.46]	0.000	0.13[0.07,0.25]	0.000
Head of household’s sex	1.14[0.94,1.38]	0.188	1.14[0.94,1.38]	0.190	1.15[0.95,1.39]	0.162
Age	1.01[1.00,1.02]	0.135	1.01[1.00,1.02]	0.137	1.01[1.00,1.02]	0.161
Informal employed	0.75[0.53,1.05]	0.0089	0.74[0.53,1.04]	0.084	0.75[0.53,1.05]	0.092
Not employed	0.70[0.45,1.08]	0.104	0.69[0.44,1.07]	0.094	0.69[0.45,1.08]	0.104
Formal employed						
Number of children	0.53[0.43,0.68]	0.000	0.54[0.44,0.67]	0.0000	0.55[0.44,0.67]	0.000
No education	0.24[0.17,0.33]	0.000	0.19[0.08,0.43]	0	0.29[0.13,0.66]	0.003
Primary school	0.40[0.31,0.52]	0.000	0.59[0.35,1.01]	0.054	0.81[0.48,1.38]	0.428
Secondary school+						
Poor women	0.38[0.31,0.47]	0.000	0.38[0.31,0.47]	0.000	0.11[0.06,0.22]	0.000
Middle wealth	0.57[0.47,0.69]	0.000	0.57[0.47,0.69]	0.000	0.28[0.14,0.56]	0.000
Rich women						
Rural*no education			1.22[0.50,2.97]	0.668	0.75[0.31,1.78]	0.512
Rural*primary school			0.60[0.33,1.08]	0.087	0.43[0.24,0.78]	0.005
Rural*secondary school+						
Rural*poor women					3.76[1.87,7.58]	0.000
Rural*middle wealth					2.08[1.01,4.30]	0.047
Rural*rich women						

**Table 6:** Logistic Regression Estimates Representing the Influence of SES on the Before Discharging Postnatal Care.

	Model 1		Model 2		Model 3	
Variables	OR [95% CI] (n = 6,924)	P- value	OR [95% CI] (n = 6,924)	P- value	OR [95% CI] (n = 6,924	P-value
Intercept	1.18[0.79,1.77]	0.411	1.11[0.73,1.69]	0.63	1.09[0.70,1.69]	0.701
Residence	0.46[0.38,0.54]	0.000	0.54[0.41,0.71]	0.000	0.55[0.40,0.75]	0.000
Head of household’s sex	1.08[0.90,1.30]	0.392	1.08[0.90,1.29]	0.421	1.08[0.90,1.29]	0.001
Age	1.02[1.01,1.03]	0.000	1.02[1.01,1.03]	0.000	1.02[1.00,1.03]	0.001
Informal employed	0.80[0.63,1.01]	0.057	0.94[0.67,1.32]	0.721	0.80[0.63,1.01]	0.06
Not employed	0.82[0.62,1.09]	0.178	0.82[0.62,1.09]	0.163	0.82[0.62,1.09]	0.164
Formal employed						
Number of children	0.48[0.39,0.59]	0.000	0.48[0.39,0.59]	0.000	0.48[0.39,0.59]	0.000
No education	0.47[0.37,0.61]	0.000	0.56[0.32,0.99]	0.045	0.58[0.34,0.98]	0.042
Primary school	0.71[0.60,0.84]	0.000	0.78[0.62,0.97]	0.024	0.78[0.64,0.97]	0.023
Secondary school +						
Poor women	0.68[0.58,0.81]	0.000	0.68[0.57,0.80]	0.000	0.66[0.48,0.91]	0.011
Middle wealth women	0.79[0.66,0.94]	0.009	0.80[0.67,0.95]	0.000	0.87[0.65,1.17]	0.362
Rich women						
Rural*no education			0.76[0.40,1.43]	0.391	0.73[0.40,1.34]	0.310
Rural*primary school			0.82[0.59,1.12]	0.210	0.81[0.59,1.11]	0.188
Rural*secondary school+						
Rural*poor women					1.06[0.73,1.53]	0.773
Rural*middle wealth					0.86[0.60,1.24]	0.416
Rural*rich women						

**Table 7:** Logistic Regression Estimates Representing the Influence of SES on the After Discharging Postnatal Care.

	Model 1		Model 2		Model 3	
Variables	OR [95% CI] (n = 7,050)	P- value	OR [95% CI] (n = 7,050)	P- value	OR [95% CI] (n = 7,050)	P-value
Intercept	0.24[0.14,0.41]	0.058	0.26[0.14,0.45]	0.000	0.27[0.15,0.48]	0.000
Residence	0.68[0.57,0.82]	0.000	0.59[0.41,0.85]	0.004	0.55[0.38,0.79]	0.001
Head of household’s sex	0.86[0.69,1.09]	0.216	0.87[0.69,1.09]	0.235	0.88[0.70,1.10]	0.257
Age	1.02[1.01,1.04]	0.001	1.01[0.99,1.04]	0.001	1.01[0.99,1.04]	0.002
Informal employed	0.75[0.58,0.96]	0.023	0.75[0.59,0.96]	0.024	0.75[0.59,0.97]	0.025
Not employed	0.90[0.67,1.22]	0.502	0.91[0.68,1.22]	0.534	0.92[0.68,1.24]	0.571
Formal employed						
Number of children	0.62[0.47,0.81]	0.001	0.61[0.47,0.81]	0.000	0.62[0.47,0.81]	0.001
No education	0.54[0.41,0.71]	0.000	0.44[0.23,0.84]	0.012	0.51[0.26,1.01]	0.054
Primary school	0.63[0.50,0.77]	0.000	0.59[0.44,0.78]	0.000	0.63[0.47,0.85]	0.003
Secondary school+						
Poor	0.69[0.55,0.86]	0.000	0.69[0.55,0.86]	0.001	0.53[0.36,0.78]	0.001
Middle wealth	0.91[0.71,1.16]	0.444	0.90[0.70,1.15]	0.410	0.84[0.57,1.23]	0.368
Rich women						
Rural*no education			1.39[0.69,2.80]	0.356	1.12[0.53,2.37]	0.771
Rural*primary school			1.20[0.80,1.79]	0.384	1.07[0.70,1.64]	0.653
Rural*secondary school+						
Rural*poor women					1.49[0.93,2.39]	0.094
Rural*middle wealth					1.12[0.67,1.88]	0.653
Rural*rich women						
